# TB survivors: why curing TB is sometimes not enough

**DOI:** 10.5588/ijtldopen.24.0530

**Published:** 2024-12-01

**Authors:** E. Pontali, R. Centis

**Affiliations:** ^1^ Department of Infectious Diseases, Galliera Hospital, Genoa, Italy;; ^2^ Servizio di Epidemiologia Clinica delle Malattie Respiratorie, Istituti Clinici Scientifici Maugeri IRCCS, Tradate, Italy.

**Keywords:** PTLD, tuberculosis, disability, pulmonary rehabilitation, quality of life

## Abstract

It is now well understood that the effect of TB does not always end when a patient completes a successful course of treatment. Successful treatment is by definition a microbiological cure, but people may continue to suffer the consequences of TB for months or years after treatment. For example, ongoing health challenges can present in the form of post-TB lung disease (PTLD), and there is a high incidence of TB-related symptoms associated with disability in other domains. We discuss an article in this issue of the *Journal* that explores the experiences of adult TB survivors, which further expands our understanding of this condition.

A patient completing TB treatment – and no longer infected with *Mycobacterium tuberculosis* – is considered a TB survivor. However, at the end of TB treatment, the lungs rarely fully recover immediately. Structural damage and functional impairment may persist for many months, years or even the rest of a person’s life – and additional structural, infectious and/or psychological complications can occur.^1^ Outside the world of TB, the phenomenon of prolonged illness after ‘cure’ will be familiar to many who have experienced the debilitating symptoms of long-COVID during recovery from COVID-19.^2^ Although TB has been with us for millennia, it is only in the past decade that reports have highlighted the persistent burden of disease in the form of post-TB lung disease (PTLD).^1,3–7^ As a consequence of this increased attention, symposia have been convened for experts to better understand the condition and to agree upon definitions of diagnosis and possible treatment for PTLD.^8,9^ Attempts at mitigating PTLD, even at the programmatic level, are being led through pulmonary rehabilitation (PR) in different settings.^3,4,10^ Clinical standards for the diagnosis and treatment of PTLD have been published, and several interventions have been proposed.^10–16^ Furthermore, for the first time, a national society, the Brazilian Thoracic Society (SBPT), has developed national recommendations for the management of PTLD,^17,18^ and the Latin American Respiratory Society (ALAT) has developed similar recommendations with a regional focus.^19^

TB survivors do not only suffer TB consequences within the lungs, but they report various degrees of symptoms and disability. In this issue of the *Journal*, McDonald et al. report on the prevalence of TB-related symptoms and self-reported disability among adult TB survivors in Uganda.^20^ This study assessed factors associated with post-TB disability using the WHO Disability Assessment Schedule v2.0 (WHODAS 2.0). This tool is useful for assessing health and disability across six domains: cognition, mobility, self-care, getting along, life activities, and social participation.^21^ In McDonald’s study, the key findings on the health status of TB survivors were as follows: 1) prevalence of post-TB symptoms was high, and 58% of TB survivors reported at least one symptom; 2) self-reported disability was even higher, with 83.5% of participating survivors reporting an issue; 3) conducting this kind of analysis using valid, consolidated tools is feasible, even in challenging settings. The study also reported results that need some consideration and further investigation. Although 70% of participants self-reported a health status ranging from good to very good, most (58%) reported one or more (16.5%) post-TB treatment symptoms. The most frequently reported symptom was cough (51%).^21^ Also contrasting with their reported good health status, the majority of TB survivors (83.5%) reported some form of disability. Disability in the mobility domain was the most common (58.5%); other frequently affected domains were cognition (42%), self-care (9.5%), getting along (30%), life activities (50%), and participation (65.5%).

The findings from McDonald et al.’s study^20^ substantially confirm the results on disability from a recent multi-country study (Kenya, Uganda, Zambia and Zimbabwe) aimed at determining disability, comorbidities and risk determinants at the end of TB treatment.^22^ In this study, it is not clear why TB survivors who experienced common TB-related symptoms, compared with whose not reporting them, were more likely to self-report some disability in one or more domains of daily functioning.^5,22–24^ Many of the problems observed could be related to the slow lung healing process, and the stigma surrounding TB (even after treatment completion) could be responsible for the social participation domain.^23,24^ Additionally, the time elapsed since treatment completion appeared to influence the self-reported disability among TB survivors. Those who had completed TB treatment within the past 6–8 months reported greater risk of disability compared to those whose treatment ended 8 or more months ago. These findings are in line with the study by Nightingale et al.,^25^ which showed that years of life lost due to disability (YLDs) decreased as the time since successful TB treatment increased.

In conclusion, in line with recent literature, the study by McDonald et al.^20^ encourages the TB community to continue to investigate early diagnosis and possible treatments for post-TB conditions.^26,27^ TB-related disability should be addressed as soon as possible after treatment completion to minimise its impact on the quality of life of TB survivors, including by expanding the continuum of care at the programmatic level. As recently commented by Calvi et al.,^28^ how TB affects people during treatment and, particularly, after successful treatment suggests that “the TB community need to look at TB as an event with the potential to profoundly derail the life trajectory of the people affected, whose management transcends the ‘simple’ boundaries of medical treatment alone.”
